# Characteristic MRI findings of the shoulder, elbow, and wrist joints in elite wheelchair basketball players

**DOI:** 10.1186/s13102-022-00528-9

**Published:** 2022-07-23

**Authors:** Masafumi Sakai, Hirotaka Mutsuzaki, Yukiyo Shimizu, Yoshikazu Okamoto, Takahito Nakajima

**Affiliations:** 1 Department of Diagnostic and Interventional Radiology, Faculty of Medicine, University of Tsukuba, 2-1-1 Amakubo, Tsukuba, Ibaraki 305-8576 Japan; 2 Department of Orthopaedic Surgery, Ibaraki Prefectural University of Health Sciences, 4669-2 Ami, Inashiki, Ibaraki 300-0394 Japan; 3 Department of Rehabilitation Medicine, Faculty of Medicine, University of Tsukuba, 1-1-1 Tennodai, Tsukuba, Ibaraki 305-8575 Japan

**Keywords:** Magentic resonance imaging, Wheelchair basketball player, Joint of upper extremity, Triangular fibrocartilage complex injury

## Abstract

**Background:**

The health of wheelchair users’ upper limbs is directly related to their quality of life. Moreover, para-sport athletes are subjected to a dual load on their upper extremities from competition and daily life, making it even more critical to maintain upper extremity health. This study aimed to investigate the characteristics of joint disorders in elite wheelchair basketball players using magnetic resonance imaging (MRI).

**Methods:**

We scanned MRI images of the bilateral shoulders, elbows, and wrist joints of ten elite wheelchair basketball players and ten general wheelchair users. The elite wheelchair players were athletes who underwent at our institution medical checkup of the candidates for the national team for the international women's tournament and who agreed to this research purpose. The general wheelchair players were recruited from wheelchair users in their 20s and 30s who had no daily exercise habits and who agreed to the study objectives. Two radiologists interpreted the MRI images and diagnosed the diseases of each joint. We compared the number of lesions between the two groups. We used Fisher's exact test to determine whether the lesions diagnosed by MRI were specific to wheelchair basketball players. The significance threshold was set at *P* < 0.05.

**Results:**

Elite wheelchair basketball players had significantly more right-sided, left-sided and bilateral latero-posterior lesions, which are cysts found on the lateral-posterior corner of the capitulum of the humerus than did general wheelchair users (*P* < 0.05). Severe damage to the right triangular fibrocartilage complex was also observed more frequently (*P* < 0.05) in wheelchair basketball players.

**Conclusions:**

We believe that the patients’ tendency to fall forward in the wheelchair hitting both hands on the ground, thereby injuring the triangular fibrocartilage complex and locking the lateral elbow, may be the cause of the characteristic findings on MRI. High-speed wheelchair operation was also considered a cause of severe triangular fibrocartilage complex injuries. This study's insights can be useful for future solutions to extend players' careers.

## Background

Wheelchair basketball is considered the most popular para-sport in the world. Wheelchair basketball players perform complex wheelchair maneuvers quickly and repeatedly, as well as playing with the ball using their upper limbs [1, 2]. In addition, they use a wheelchair in their daily lives [3, 4], so their upper limbs are doubly stressed.

Therefore, maintaining the health of the upper extremities of wheelchair basketball players significantly impacts their careers and quality of life (QOL) [5, 6].

Recent studies have shown that wheelchair users have unique abnormal findings in the shoulder, elbow, and wrist joints on MRI [7–9]. Therefore, it is highly likely that an MRI of the upper extremity joints of wheelchair basketball players will show more complex abnormal findings. If the characteristics of the injury can be determined, it will be possible to identify actions to avoid damage. This information will be beneficial for elite wheelchair basketball players in extending their lifespan.

The purpose of this study was to perform bilateral MRI examinations of the shoulder, elbow, and wrist joints of elite-level wheelchair basketball players to investigate the characteristics of MRI images of the upper extremity joints, and to examine whether there are any differences between the findings of wheelchair basketball players and those of general wheelchair users based on the previous literature [7–9]. The results of this study can be applied to the prevention of disabilities in other wheelchair sports and the maintenance and improvement of the QOL of people in wheelchairs. In this case, we believe that para-sports will become even more popular.

## Methods

### Subjects

Ten elite wheelchair basketball players and ten general wheelchair users participated in this study. The medical checkup of the candidates for the national team for the international women's tournament was conducted at our institution, and ten female elite players who agreed to this research purpose were included in the study.

The general wheelchair users included patients who presented to our institution between 2018 and 2021. They were wheelchair users in their 20s and 30s with no daily exercise habits, and had agreed to the study objectives.

The exclusion criteria for both groups were using a power wheelchair and having used a wheelchair for less than 6 months. All participants were scanned from November 2018 to November 2021. The study was approved by the Ethics Committee of our institution. The approval date was August 13, 2018. Informed consent was obtained from the study participants in writing prior to the study. If written informed consent was not obtained, it was obtained in the form of opt-out on the website.

### MRI scan

MRI of the right shoulder, right elbow, right wrist, left shoulder, left elbow, and left wrist was performed on all participants. The machine used was 1.5 T MRI (Vantage XG 1.5T, Cannon, Japan).For details of the imaging parameters, a previous paper is referred to [7].

### Imaging interpretation

MRI interpretations were performed by two radiologists. One was a musculoskeletal radiologist with 18 years of experience. The other was a general radiologist with 8 years of experience. The radiologists interpreted the images independently and blindly. In interpreting MRI of the bilateral shoulder, elbow, and wrist, reproducibility tests were performed by two radiologists. And agreement rates and kappa values were calculated (Tables 1, 2, 3). In the interpreting, neuropathy-related diseases (e.g., carpal tunnel syndrome) were not considered significant findings. This is because these findings on MRI are often not symptomatic.

**Table 1:** Shoulder MRI findings in elite basketball players and general wheelchair users, number of people with shoulder symptoms, and results of repeatability test

Shoulder injury	Wheelchair basketball players				General wheelchair users				Inter-observer agreement rate (%)	Kappa coefficient
	R	L	Bi	With symptom (R/L)	R	L	Bi	With symptom (R/L)		
Partial tear of supraspinatus tendon	4	3	2	2/1	1	0	0	0/0	81	0.63
OA of gleno-humeral joint	1	0	0	0/0	0	0	0	0/0	82	0.88
Superior labral tear	0	1	0	0/0	0	0	0	0/0	91	0.91

MRI, magnetic resonance imaging; OA, osteoarthritis; R, right; L, left; Bi, bilateral

**Table 2:** Elbow MRI findings in elite basketball players and general wheelchair users, number of people with elbow symptoms, and results of repeatability test

Elbow injury	Wheelchair basketball players				Gneneral wheelchair users				Inter-observer agreement rate (%)	Kappa coefficient
	R	L	Bi	With symptom (R/L)	R	L	Bi	With symptom (R/L)		
LP lesion	7*	6*	5*	1/2	2	1	0	0/0	93	0.72
OA	0	0	0	0/0	2	0	0	0/0	83	0.66
MCL injury	0	0	0	0/0	0	1	0	0/0	93	0.86

MRI, magnetic resonance imaging; LP, latero-posterior; OA, osteoarthritis; MCL, medial collateral ligament; R, right; L, left; Bi, bilateral

* indicates *P* < 0.05

**Table 3:** Wrist MRI findings in elite basketball players and general wheelchair users, number of people with wrist symptoms, and results of repeatability test

Wrist injury	Wheelchair basketball players				Generalwheelchair users				Inter-observer agreement rate (%)	Kappa coefficient
	R	L	Bi	With symptom (R/L)	R	L	Bi	With symptom (R/L)		
Severe TFCC injury	5*	2	2	2/1	0	0	0	0/0	92	0.81
Minor TFCC injury	2	0	0	0/0	3	2	2	0/0	77	0.55
Extensor carpi ulnaris tendon tear	0	0	0	0/0	0	1	0	0/0	95	0.66

MRI, magnetic resonance imaging; TFCC, triangular fibrocartilage complex; R, right; L, left; Bi, bilateral

* indicates *P* < 0.05

### Definition of abnormal findings

We defined abnormal findings on MRI as shown in Table 4.

**Table 4:** Definition of abnormal findings on MRI

Disease	Definition of abnormal findings on MRI
Tear of tendon	High signal intensity in the tendon on T2-weighted image or proton density weighted image
Osteophytes	Bony humps around joints
Subchondral cyst	Cyst formation just below the articular cartilage
Injury of fibrocartilage (e.g., triangular fibrocartilage complex injury)	High signal area in fibrocartilage on some sequences with or without swelling
Injury of ligament	High signal area in a ligament on some sequences with or without swelling

MRI, magnetic resonance imaging

### Grading of diseases

Rotator cuff tears were classified into two groups according to severity. A partial tear was defined as an abnormal signal intensity within the rotator cuff, while a full-thickness tear was defined as an abnormal signal intensity extending from one end of the rotator cuff to the other.

We graded the injury of the triangular fibrocartilage complex (TFCC) into two groups. Minor injury was defined as damage to one or two components of the TFCC, and severe injury was defined as damage to the entire TFCC.

### Definition of the latero-posterior lesion

We defined the latero-posterior (LP) lesion as cysts of various sizes found on the lateral-posterior corner of the capitulum of the humerus. The differentiating features from the subchondral cysts were the site of predilection and the presence of subchondral bone between the cartilage and the cyst.

### Analysis

The number of lesions at each joint was compared between two groups: elite wheelchair basketball players and general wheelchair users. Fisher's exact test was used, with a significance threshold of *P* < 0.05.

## Results

This study included ten elite wheelchair basketball players, all of whom were women with a mean age of 29.6 years (SD: ±6.9). There were also ten general wheelchair users in the control group: nine men and one woman; the mean age was 30.1 years (SD = ±7.8).

The mean history of wheelchair use among elite wheelchair basketball players was 14.2 years (SD = ±4.9). The control group of general wheelchair users was 13.1 years (SD = ±2.1). No significant difference was found between the two in the Mann–Whitney U test. Elite wheelchair basketball players had a mean athletic career of 11.6 years (SD = ±4.6).

The underlying diseases of the elite para-athletes were skeletal and neurological diseases, such as spinal cord injuries, in seven and three patients, respectively, whereas in general wheelchair users, there were five and five users, respectively.

The scanning time was 2 h 25 min for elite wheelchair basketball players and 2 h and 22 min for general wheelchair users. The top three disease names and the number of patients with findings in the shoulder, elbow, and wrist joints on MRI images of elite wheelchair basketball players and general wheelchair users are summarized in Tables 1, 2 and 3. The patients in whom abnormal MRI findings matched the patient's chief complaint are also listed in Tables 1, 2 and 3.

In the shoulder joint, the most frequent abnormality was supraspinatus tendon injury. Superior labral injury and osteoarthritis of the glenohumeral joint were also observed in one patient each. There was no significant difference in the number of these findings between elite wheelchair basketball players and general wheelchair users.

In the elbow joint, the top three most frequent abnormalities were LP lesions, osteoarthritis, and medial collateral ligament injury. Of these, right LP lesions were found in seven, left LP lesions in six, and bilateral LP lesions in five elite wheelchair basketball players, with a statistically significant difference compared to general wheelchair users (*P * < 0.05).

In the wrist joint, the top three most frequent abnormalities were severe TFCC injury, minor TFCC injury, and ulnar extensor carpal tendon injury. Of these, right severe TFCC injuries were more common in elite wheelchair basketball players, with a statistically significant difference of five in the elite wheelchair basketball players and zero in the general wheelchair users (*P * < 0.05). Interestingly, the number of patients with abnormal MRI findings that matched the chief complaint was not very large.

## Discussion

**Fig. 1:**
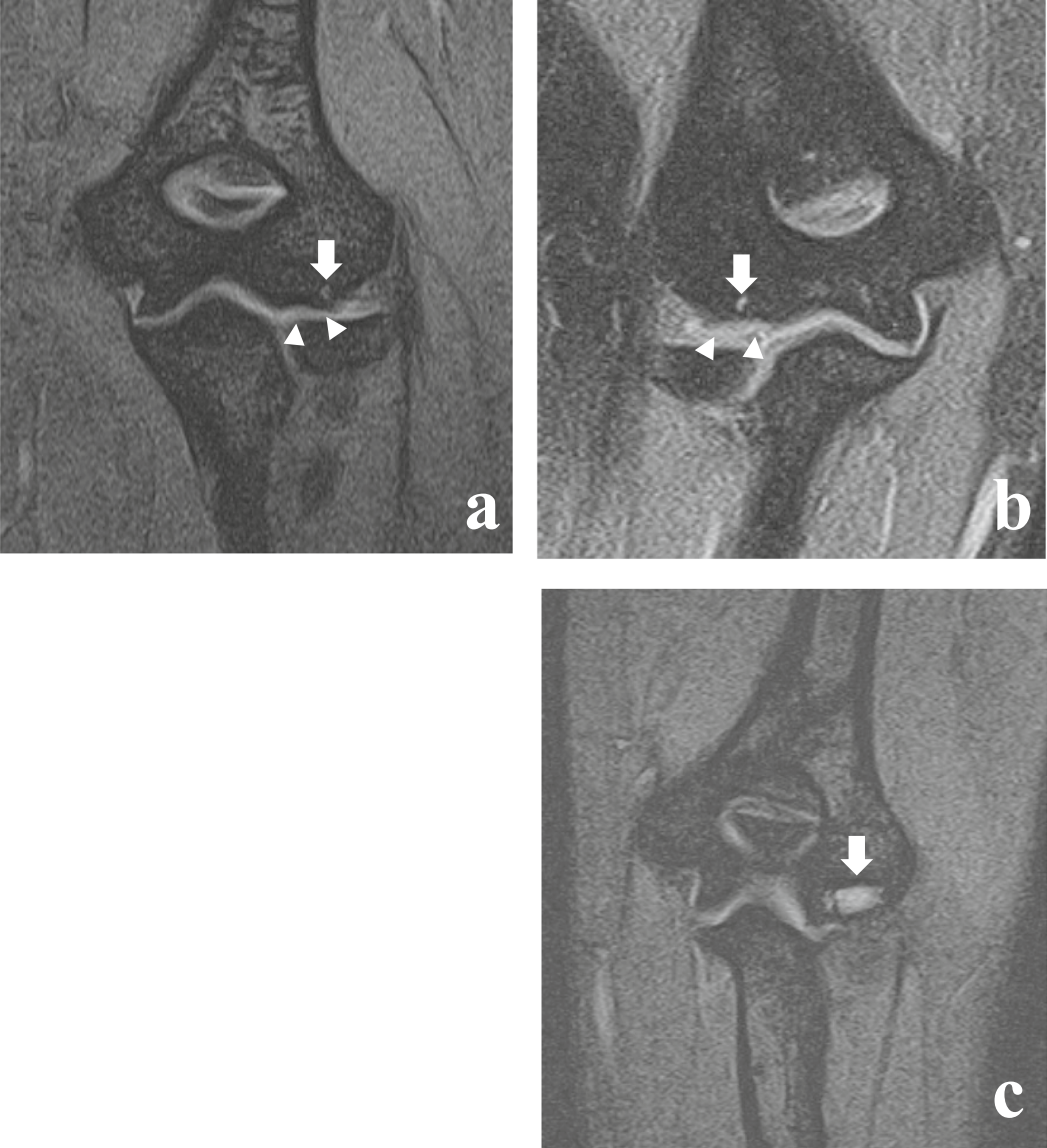
MRI finding of various LP lesions. **a**, **b** A 32-year-old woman with bilateral LP lesions, which appeared as a small cyst at the LP part of the capitulum (arrows) in the left (**a**) and right (**b**). The arrowheads suggest that cartilage is observed, and cysts exist in the subchondral bone. These findings suggest that the entity of this cyst is apparently different from the subchondral cysts observed in osteoarthritis. **c** A 27-year-old woman with a LP lesion, which appears as a large, flat-shaped cyst with a septum (arrow) at the LP part of the capitulum. Image was acquired by T2* weighted imaging

This study revealed a tendency for elite wheelchair basketball players to exhibit multiple characteristically abnormal findings in their bilateral upper extremity joints. First, bone cysts of various sizes were more likely to occur in the lateral-posterior region of the elbow joint. This is consistent with what was reported as an LP lesion in a previous study [7, 10], and is therefore included in the results as LP lesions in the text (Fig. 1). In addition, the TFCC was easily injured. No significant findings were observed in the shoulder joints.

A previous study showed that the frequency of LP lesions and TFCC injuries is high among general wheelchair users [7]. The average age of the subjects in the previous study was approximately 50 years [7]. On the other hand, the mean age of the subject group of the general wheelchair users in the present study was 30.1 years, and there were no significant abnormal findings. Therefore, it is suggested that the frequency of abnormal findings in the upper extremities of general wheelchair users increases with age.

The elite wheelchair basketball players, as young as 29.6 years old, showed remarkable abnormal findings. These findings suggest that LP lesions and severe TFCC injuries are characteristic upper limb joint findings in elite wheelchair basketball players.

Although these LP lesions and severe TFCC injuries have been observed in general wheelchair users, previous literature suggests that the cause is not excessive wheelchair operation, but frequent push-up movements performed to prevent pressure ulcers over several years [7, 11, 12]. The push-up motion must be performed many times a day by wheelchair users, and presumably causes TFCC injuries and LP lesions because the load is applied to the wrist joint by the forearm axis with locking of the elbow joint [7, 13, 14].

Because elite wheelchair basketball players are still young, severe TFCC injuries and LP lesions are likely not only caused by push-ups but also by the competitive characteristics of wheelchair basketball.

Although they handle the ball all the time, passing, shooting, and dribbling were not unnatural and overloaded movements for the wheelchair basketball players in this study. Therefore, it is unlikely that playing basketball produces characteristic findings for elite wheelchair basketball players.

**Fig. 2:**
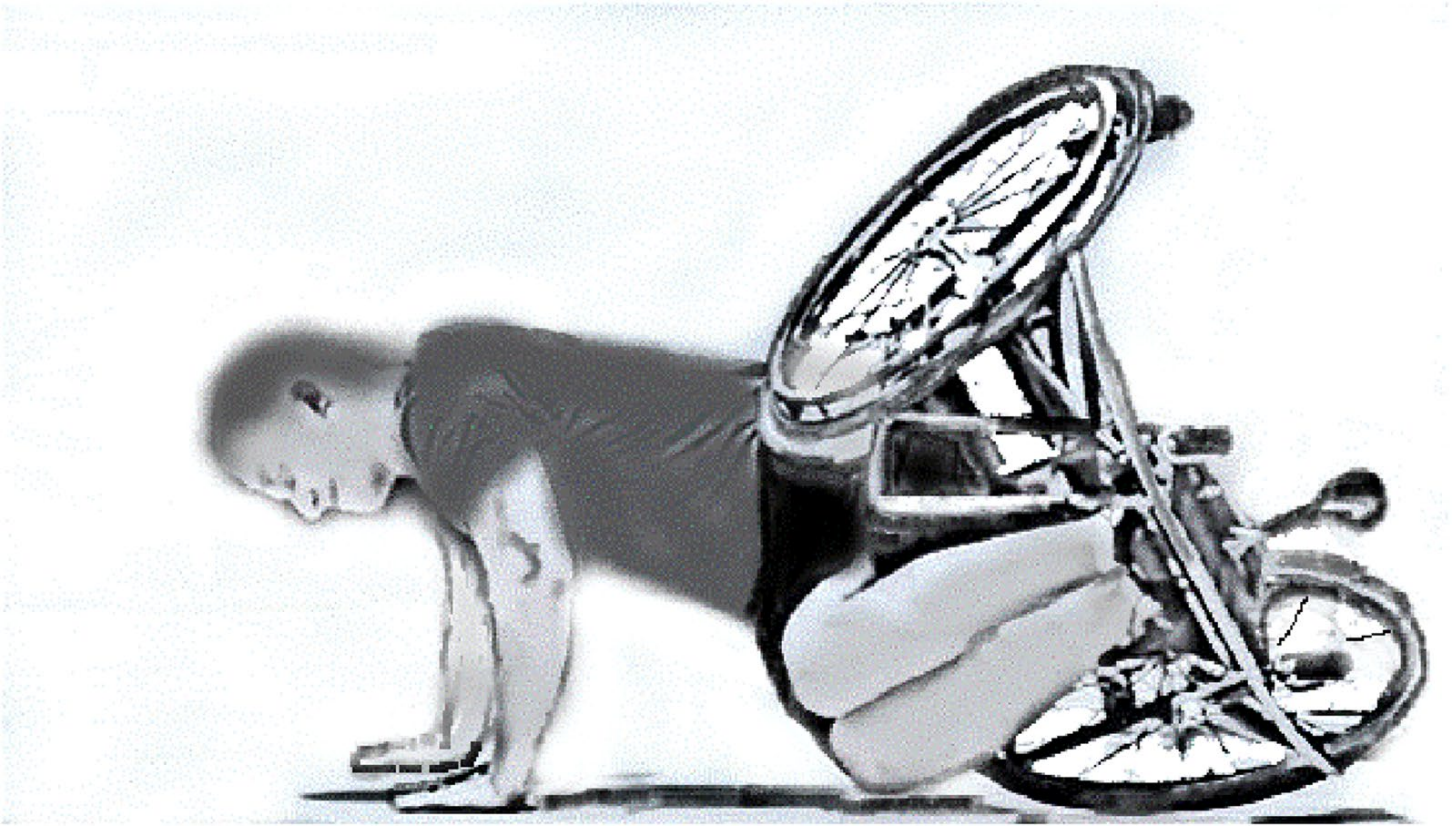
Illustration of a wheelchair basketball player falling forward while playing. The illustration shows a player falling forward with a wheelchair during the game. At the time, players tend to fall on to the hands bilaterally. In this situation, the hands are on the ground with the elbows extended. Therefore, the load is applied to the wrist joint by the forearm axis with locking of the elbow joint, causing TFCC injury and locking of the elbow at the lateral side. This illustration is an original drawing by MS, the co-author, based on a photo of a player falling forward in an actual wheelchair basketball game

Compared to other wheelchair sports, wheelchair basketball is characterized by a high incidence of “tipping the wheelchair forward” [15]. For example, as shown in Fig. 2, when a player makes a layup shot, the upper body is often thrown out of the wheelchair, and the wheelchair tends to fall forward [15]. There are also many other cases in which the wheelchair falls forward during the game [15]. This is because the player's body is fixed to the wheelchair to some extent. As shown in Fig. 2, when a wheelchair falls forward, it is most common for the player to fall on the hands and elbows to avoid hitting the face or twisting the neck. If a person falls with his or her hands, the TFCC is overloaded [16]. If the elbows are locked, the load on the TFCC is even greater [16]. In addition, when the elbow is locked, it is hyperextended at the time of the fall, which increases the likelihood of the load being applied to the lateral side of the elbow. Severe TFCC injuries and LP lesions are assumed to occur under these circumstances. In addition, keeping hands on the wheelchair from 6 o'clock direction to 9 o'clock direction when driving the wheelchair at full force is also considered a risk for severe TFCC injury. Motion analysis using motion capture may further clarify the cause of the problem, which will be the subject of future research.

The forward fall of a wheelchair is an attractive element for watching wheelchair basketball games and is difficult to avoid from a competitive standpoint.

Falling on the elbow joint or forearm, for example, may not produce the present result, but it is not recommended because it requires training to change the way of falling and may cause further injury.

Moreover, wearing a supporter on the hand or elbow may change the outcome, and can affect the performance. We believe that these issues require further interventional research.

In summary, the characteristic MRI findings of the upper extremities in elite wheelchair basketball players are severe TFCC injuries and LP lesions. These lesions are thought to be caused when players fall with their hands in front of the wheelchair, lock their elbow joints during the fall, and keep their hands on the wheel from 6 to 9 o'clock while driving the wheelchair at full speed.

An interesting finding of this study is that there was not a large agreement between the abnormal imaging findings and the chief complaint among both elite wheelchair basketball players and general wheelchair users. The reason for this is unclear, but we speculate that the upper body muscles of wheelchair users are generally well developed compared to those of healthy people. This results in improved joint stabilization and, consequently, less pain. In addition, the abnormal findings in this paper reflect the soft-tissue damage caused by frequent falls. A single trauma does not cause much damage; however, repeated trauma associated with similar falls may result in histological damage, even if the patient does not feel pain. Therefore, we assume that the patient did not complain of pain, but exhibited abnormalities on imaging.

This study had two limitations. First, only ten elite wheelchair basketball players were scanned; however, the number of elite wheelchair basketball players is small, and opportunities to gather as a group outside of training camps are limited. In the future, we will consider the use of a mobile MRI system to clarify this problem [17]. In addition, while all elite wheelchair basketball players were women, most of the general wheelchair users were men.

The incidence of spinal cord injury is reportedly higher in males worldwide [18]. The latest epidemiology in our country also shows that spinal cord injuries are more common in men [19]. In addition, head and neck trauma is reportedly more common in men at younger ages [20]. These may be the biggest reason why it was difficult to gather female wheelchair users in this study. Furthermore, as already mentioned, MRI of the upper extremity joints of general wheelchair users may be influenced by push-up as a basic life factor in the background [7]. There is no difference between men and women in this regard [7]. Therefore, we assume that the influence of sex differences on the results of this study is small.

## Conclusion

In a previous study, characteristic MRI findings were observed in the upper limb joints of wheelchair users, and it was speculated that “push-up” to prevent pressure ulcers, a movement unique to wheelchair users, might have caused these findings [7].

Our study revealed characteristic MRI findings of upper extremity joints in elite wheelchair basketball players, where LP lesions and severe TFCC injuries were observed. These findings were considered to be mainly due to the characteristic movements associated with wheelchair basketball, such as falling forward with the wheelchair during competition and high-speed wheelchair handling.

## Data Availability

The datasets used and/or analyzed during the current study are available from the corresponding author on reasonable request.

## Abbreviations

QOL: Quality of life
MRI: Magnetic resonance imaging
TFCC: Triangular fibrocartilage complex
LP lesion: Latero-posterior lesion
SD: Standard deviation
OA: Osteoarthritis
MCL: Medial collateral ligament
